# The Composite Insect Trap: An Innovative Combination Trap for Biologically Diverse Sampling

**DOI:** 10.1371/journal.pone.0021079

**Published:** 2011-06-16

**Authors:** Laura Russo, Rachel Stehouwer, Jacob Mason Heberling, Katriona Shea

**Affiliations:** 1 Biology Department, Pennsylvania State University, University Park, Pennsylvania, United States of America; 2 Department of Landscape Architecture, University of Michigan, Ann Arbor, Michigan, United States of America; 3 Department of Biology, Syracuse University, Syracuse, New York, United States of America; University of Otago, New Zealand

## Abstract

Documentation of insect diversity is an important component of the study of biodiversity, community dynamics, and global change. Accurate identification of insects usually requires catching individuals for close inspection. However, because insects are so diverse, most trapping methods are specifically tailored to a particular taxonomic group. For scientists interested in the broadest possible spectrum of insect taxa, whether for long term monitoring of an ecosystem or for a species inventory, the use of several different trapping methods is usually necessary. We describe a novel composite method for capturing a diverse spectrum of insect taxa. The Composite Insect Trap incorporates elements from four different existing trapping methods: the cone trap, malaise trap, pan trap, and flight intercept trap. It is affordable, resistant, easy to assemble and disassemble, and collects a wide variety of insect taxa. Here we describe the design, construction, and effectiveness of the Composite Insect Trap tested during a study of insect diversity. The trap catches a broad array of insects and can eliminate the need to use multiple trap types in biodiversity studies. We propose that the Composite Insect Trap is a useful addition to the trapping methods currently available to ecologists and will be extremely effective for monitoring community level dynamics, biodiversity assessment, and conservation and restoration work. In addition, the Composite Insect Trap will be of use to other insect specialists, such as taxonomists, that are interested in describing the insect taxa in a given area.

## Introduction

There are many methods of insect collection, both active and passive. However, most specialize on one type of insect and depend on the insect's behavioral response to stimuli. This is ideal for studies that focus on a single taxon or guild of interest, but these traps do not collect a representative sample of all insects present in a given ecosystem. In fact, many scientists recommend using multiple trap types to ensure a complete collection, even for just one taxon [1]–[5]. However, it is time consuming and expensive to implement multiple trap types.

Because we were interested in the diversity of the flying insect community rather than in one particular taxon, none of the traditional trap types were sufficient by themselves, and it would have been prohibitively expensive to use multiple trap types. For these reasons, we designed a nonspecific insect trap, the Composite Insect Trap. To construct this passive trap, we incorporated the design of components from other, more targeted trap designs. In addition to the collection of a diversity of insect taxa, we designed the Composite Insect Trap to be cost-efficient and easy to assemble and disassemble, making it ideal for rapid biodiversity assessment, investigative pilot studies, large-scale censuses, low-budget research, and educational purposes. We also designed the Composite Insect Trap to be easy to transport so that we could move it long distances between trapping locations, and robust so that it would withstand inclement weather conditions.

Our trap was inspired by four other widely used and well recognized passive trapping devices for catching flying insects: the malaise trap, cone trap, pan trap, and flight intercept trap [1], [6]–[9]. Each of these trap types has advantages and disadvantages and there are many cases where one of these traps excels while another is deficient (e.g. [10]). The deficiencies of a trap are particularly restrictive when insect biodiversity is of interest, because they will fail to catch select groups of insects. Here we briefly describe each of these traps and how they influenced the construction of the Composite Insect Trap.

Malaise traps catch those insects that fly upward to avoid an obstruction in their flight path [11]. They are very widely used and typically involve a mesh netting canopy which slopes up, forcing insects into a collecting jar filled with a killing agent. Malaise traps have been shown to be consistent and reliable in the species they catch over a summer [12].

Cone traps (specifically “Texas” cone traps) consist of a wire mesh cone on a pole with a collection container on top and also catch insects that fly upward. However, the cone trap differs from the malaise trap in that it employs a bait to attract insects [13]. Although there are many studies touting the effectiveness of the cone trap, it is inherently species-specific because the bait is often pheromone based, and is thus unlikely to attract nonfocal species. Cone traps are usually used to monitor Lepidopteran pests, such as the tobacco budworm [13], [14].

Flight intercept traps consist of a vertical mesh barrier above a collection dish filled with a killing agent. The flight intercept trap catches those insects that drop down to avoid obstacles. Although one study found that malaise traps caught a greater diversity and abundance of beetles than flight intercept traps [12], most studies agree that beetles drop when encountering an obstacle and are more likely to be caught in a flight intercept trap [1], [15], [16]. As with malaise traps, the mesh barrier intercepts not only local arthropods, but also those that are dispersing through an area and is thus an important component of the trap, allowing it to capture more than the collection dish would alone [3]. This trap is sometimes constructed with plastic or glass instead of mesh netting, but these materials not only make the trap more expensive, but also heavier, bulkier, and more susceptible to damage [15].

Pan traps consist of a shallow dish filled with soapy water. The soap acts as a surfactant and breaks the surface tension so that the insects drown and can be collected later. Pan traps are often painted to be attractive to various kinds of insects. In particular, yellow pan traps [6] are broadly attractive to pollinators, aphids, and parasitoid wasps [3]. Although certain groups are more attracted to blue pan traps, yellow seems to attract a greater diversity of insects [17], [18]. Other insects are attracted to the pan trap because of the water. In some cases, pan traps have been shown to collect groups that are poorly represented by malaise trap collections and sometimes are more effective at catching pollinators [2], [19].

**Figure 1:**
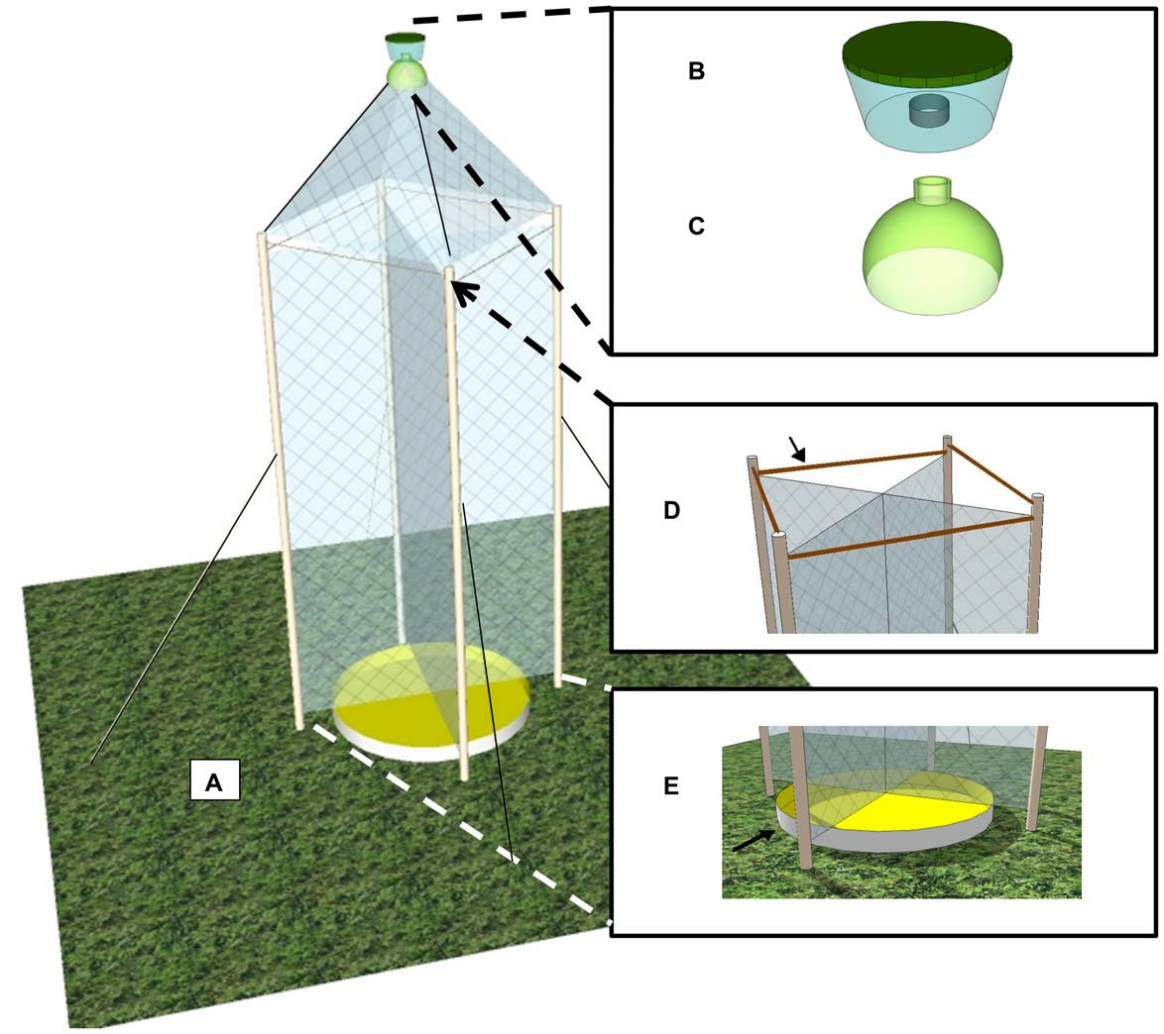
The Composite Insect Trap. **A**) The Composite Insect Trap is a passive trap designed to collect as broad a spectrum of insects as possible, utilizing ideas from other, more specialized trap designs in a novel construction. The plastic components of the cone form the collection chamber (B and C). **B**) The modified plastic container with embedded bottle top will hold the alcohol for killing and storing insects. **C**) The top of the two-liter bottle will be attached to the mesh fabric of the cone. **D**) The bamboo rods stabilize the top part of the flight intercept trap. **E**) The yellow pan trap sits below the flight intercept trap and is filled with soapy water to act simultaneously as a killing and collecting dish.

The Composite Insect Trap has components that resemble each of these standard trap types (Fig. 1). The cone portion of the Composite Insect Trap combines elements from the malaise and cone traps. Although it superficially resembles the cone trap in appearance, it is functionally more closely related to the malaise trap because it relies on the malaise method of forcing the insects upward to avoid a mesh obstruction below and has a collecting container filled with a killing agent instead of a pheromone bait. The cone component of the Composite Insect Trap serves as the capturing mechanism and is set on top of the middle portion of the Composite Insect Trap, a flight intercept trap that captures insects that fly downward as well as upward. As a collection dish below the flight intercept trap, there is a yellow pan trap filled with soapy water that collects insects attracted both by the yellow color and by the water, as well as those that drop down when encountering the mesh of the flight intercept component.

By combining these designs, we aim to maximize the advantages of each trap type and minimize their disadvantages without requiring the collector to set up multiple traps at every sampling site. Our Composite Insect Trap is designed so that the deficiencies of each component are covered by the others. Although the Composite Insect Trap also has biases, as dictated by its components, it is designed to capture the broadest diversity of insects, and to be as nonspecific as possible. The Composite Insect Trap is not tailored to collect any one taxon and provides a nonspecialized method to collect a diversity of insects, thus providing an alternative that reduces the cost in a sampling design targeted at biodiversity assessment.

## Materials and Methods

In the Composite Insect Trap, elements of the malaise trap design are used in the cone and flight intercept trap components, but there is not a separate malaise component. Thus, the trap has three main components: cone, flight intercept, and pan traps (Fig. 1A). Each of these components is constructed separately and then assembled at the field site.

### Cone trap construction

We used readily available components to build the Composite Insect Trap (Table 1). To prepare the collection chamber of the cone portion of the trap, we cut the top of a two-liter bottle (PET plastic) approximately 6 cm down (where the curve began) (Fig. 1C). The lid of the two-liter bottle also needed modification. We removed the top of the lid so that only the threads remained, thus creating a tube which could connect the collection chamber to the cone section. Although the lid of the two-liter bottle and the plastic container became one unit, it was still possible to attach the lid to the top of the bottle, while leaving an opening for insects to fly through.

**Table 1:** Components required to build the three portions of the Composite Insect Trap.

Materials Needed:	Cone	Flight Intercept	Pan
Strips of canvas fabric	X		
4 PVC flags (with flags removed)	X		
1 plastic container with lid (∼11.5 cm diameter and ∼5.5 cm deep) for holding alcohol	X		
1 two-liter soda bottle (PET plastic) with lid	X		
Silicone sealant (GE Silicone II Kitchen and Bath)	X		
70% ethanol	X		
Mesh netting (bridal tulle)	X	X	
String	X	X	
Hem tape		X	
4 thin bamboo rods		X	
4 PVC pipes 2 cm diameter, 1.5 m length		X	
4 plastic garden stakes		X	
1 drill with drill bits		X	
4 plastic rebar stakes		X	
Soap			X
Water			X
1 aluminum pan (34×46 cm, ∼9 cm deep)			X
Yellow spray paint, “Rust-oleum” gloss protective enamel			X

Next, we modified a plastic container (Fig. 1B). We cut a hole in the center of the bottom of the container and inserted the lid of the two-liter bottle until the bottom of the lid and the bottom of the container were level. The top of the lid now protruded into the empty container (Fig. 1B). The lid was held in place by a sealant applied around the edge of the lid/container connection. When filled with alcohol and attached to the severed top of the two-liter bottle, this formed the collection chamber.

**Figure 2:**
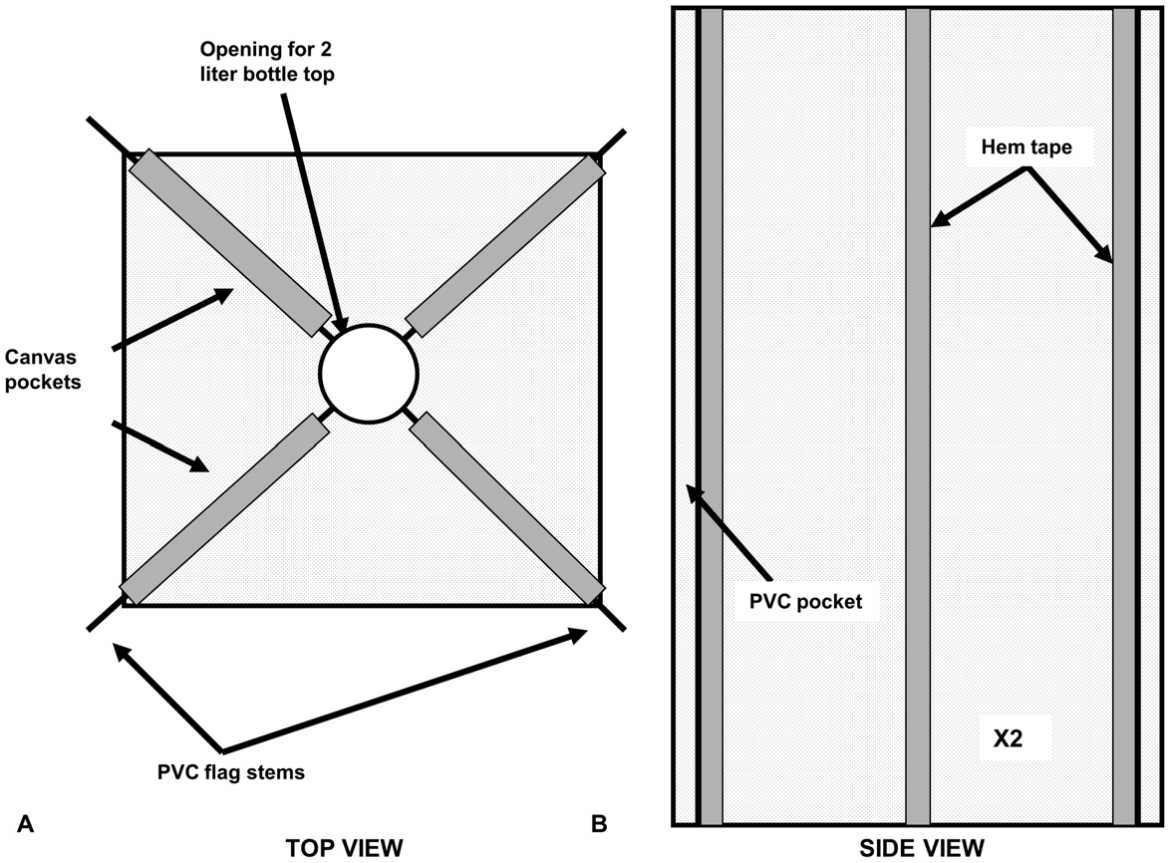
Mesh components of the Composite Insect Trap. Mesh fabric (bridal tulle) is represented by the dotted areas. **A**) The mesh cut out for a cone trap. Four canvas pockets (solid) are sewn onto the diagonals and contain the PVC flag stems. **B**) The mesh cut out for a flight intercept trap. Two of these fabric sections are sewn together along the center line, which is reinforced with hem tape. The pockets for the PVC pipes are along the sides and are also reinforced with hem tape.

The collection chamber was then attached to a cone constructed from a square piece of mesh netting, approximately 80 cm to a side. We sewed canvas strips (approximately 50 cm) onto the mesh using a sewing machine to create pockets that held the PVC flag stems (Fig. 2A). A hole was cut out of the center of the mesh to create a narrow opening at the top, which was attached with silicone sealant onto the collection chamber.

In use, the container at the top of the trap was filled with 70% ethanol to just below the rim of the bottle cap. Insects flew in through the hole in the bottle cap and were simultaneously killed and preserved in the ethanol.

### Flight intercept construction

To build the flight intercept portion of the trap (Table 1), we first drilled one hole through each PVC pipe, approximately one centimeter from the top, and a second hole another centimeter below and offset by 90 degrees to the first. In the field, we inserted bamboo rods through these holes to stabilize the trap. We also drilled a small hole approximately 75 cm from the bottom of the pipe.

The flight intercept trap required two rectangular pieces of mesh netting (approximately 120 cm long and 90 cm wide), which were attached along the center in an “X” pattern. Using a sewing machine, we sewed hem tape along the longitudinal center line of each piece of mesh and then sewed the hem tape strips together to connect the pieces. Next, we created pockets large enough to hold the PVC pipes by folding over the side of the mesh and sealing it with hem tape to avoid tearing (Fig. 2B).

After placing the pipes in the mesh pockets, we threaded the string through the hole 75 cm from the bottom of the pipe. We then tied the other end of the string to the garden stake and repeated this procedure for the three other pipes.

To set the trap up at a site, we stood the PVC pipes on top of the four plastic rebar stakes, which were set approximately a half meter apart in the ground. We then pulled the string taut so that the pipes stood upright at each of the four corners, and hammered the garden stakes into the ground. Finally, we threaded the bamboo rods through the holes at the top of the pipes to stabilize the trap (Fig. 1D).

### Pan trap construction

The pan trap was simple to construct (Table 1). We sprayed yellow paint (“Rust-oleum” gloss protective enamel, yellow) on the inside of an aluminum pan 34 by 46 and 9 cm deep. In use, we filled the pan with soapy water and placed it beneath the flight intercept trap (Fig. 1E).

### Field procedure

We designed, constructed, and utilized six Composite Insect Traps during the summer of 2009. The traps were in continuous use from June through August and were set up, taken down, and transported between field sites on a daily basis. Over the summer, these six traps were set up a total of 134 times; 90 times in an agricultural system and 44 times in weedy fallow fields in central Pennsylvania, USA. Traps were left up for 24 hours for each collection event.

During collection, the chamber of the cone trap with its ethanol and insect specimens was emptied into a labeled scintillation vial. The pan trap was emptied through a funnel into a mesh bag, which was then stored in the freezer until it could be processed.

### Processing procedure

We identified the insect specimens collected in the traps by following the dichotomous keys provided in Borror and DeLong's *Introduction* *to the Study of Insects*, 7^th^ edition [20]. We tested the differences in mean abundance of insects caught per trap per day and mean number of orders captured per trap per day between the cone and pan traps with a two-tailed *t* test. Voucher specimens of the insects collected will be stored at the Pennsylvania State University.

## Results and Discussion

Insects are a hyperdiverse taxonomic group, probably more diverse than any other terrestrial metazoan group [21], [22]. There is a need for new collection methods that are cost effective, precise, and reliable in order to document the taxonomic and ecological value of this important class of animals. We know very little about insect diversity; it is estimated that less than 20% of the species on Earth have been identified and described [23], but this estimate is based entirely on the diversity of small samples of tropical arthropods [24], [25]. Equipment similar to the Composite Insect Trap would be useful for consistent sampling across multiple taxa and for documenting and understanding the diversity of insects. Toward this goal, we designed the Composite Insect Trap to be nonspecific and to collect a range of taxa. It is a composite of four commonly used trap types, and collects insects that avoid obstacles by flying upward, as well as those that avoid obstacles by dropping down. To the best of our knowledge, no one else has published a description of a similar trap, although unpublished designs may exist.

The Composite Insect Trap is a useful tool for sampling at multiple locations, as it can be assembled and disassembled rapidly and transported easily. It is light weight but withstands weather well and can be used for multiple years. A complete trap weighs approximately 4.5 kg, excluding the alcohol and soapy water required to fill the cone and pan components, respectively. One person was able to set up the trap alone and required approximately 15 min to assemble or disassemble a Composite Insect Trap at a sampling location. Despite multiple thunderstorms and high winds, the traps never collapsed or were destroyed. Minor damage, such as tearing, was easily repaired with patches of mesh netting. If a trap sustains more severe damage, its individual components are affordable and easy to replace. In terms of durability, the Composite Insect Trap is similar to malaise traps in that the fabric becomes more fragile with time due to exposure to UV light (J. Tooker, personal communication). However, at the end of the summer, the traps were in good condition for use in future field seasons.

We collected almost 15,000 specimens of 21 different orders with the Composite Insect Trap over a period of three months during the summer of 2009. All of these specimens were identified to the order level. At this resolution, we found great diversity. The majority of the insects in the traps were Diptera (56%), Hemiptera (26%), Coleoptera (7%), and Hymenoptera (7%), but there were representatives from the insect orders Blattodea, Collembola, Dermaptera, Ephemeroptera, Lepidoptera, Mecoptera, Neuroptera, Odonata, Orthoptera, Plecoptera, Psocoptera, Thysanoptera, Trichoptera, and non insect arthropods such as Acari (mites), Araneae, Opiliones, and Diplopoda.

Because the pan and cone components of the Composite Insect Trap were collected in separate chambers, their captures could be evaluated separately. However, their captures were not independent of each other because they shared the trapping mesh of the flight intercept component. The pan component caught a greater abundance of insects and a greater diversity of insect orders (abundance per trap day [mean, SE]: cone [4.34, 0.50], pan [106.03, 5.56], *P*<0.001; number of orders: cone [1.57, 0.10], pan [5.44, 0.13], *P*<0.001). Despite the efficacy of the pan trap, Neuroptera was only found in the cone component and, at a higher taxonomic resolution, it may be found that the cone component selectively catches some taxa the pan does not. Compared to standard “Texas” style cone traps, which are designed to catch a particular pest organism, the cone component of the Composite Insect Trap nonetheless catches a much greater variety of insects [13].

In order to fully test the efficacy of the Composite Insect Trap, we would ideally have deployed a full suite of other insect traps at the same sampling sites for comparative purposes. Unfortunately, this was financially and logistically prohibitive. Instead, we compared our collection to published studies using a range of other trap types. The Composite Insect Trap appears to compare well with these studies in terms of the number of taxa collected [3], [4], [16], [18]. Even though these studies took place in a broad range of habitats, from salt marsh [18] to tropical forests [4] to agricultural landscapes [3], the Composite Insect Trap lacked only 3 insect orders that were caught in these other studies: Mantodea, Isoptera, and Archaeognatha. It is unknown whether the absence of these orders was due to their actual distribution or to a bias of the trap itself.

For a researcher interested in the biases of the trap, it would be possible to test the Composite Insect Trap against the other trap types. It would also be possible to achieve a greater understanding of the sampling biases of the Composite Insect Trap by comparing catches with and without different components. For example, one could compare the catch in the pan with and without the yellow paint. Finally, it would be possible to test different colors or sizes of mesh to understand the visibility of the material used in the flight intercept and cone components. All traps have intrinsic biases, however, and the biases of the Composite Insect Trap should not prevent it from being a useful tool in studies focusing on insect diversity.

Although it is difficult to compare the results of our study directly with those in published studies because of differences in climate, habitat, and sampling design, the diversity collected by the Composite Insect Trap appears to compare favorably at the level of insect order with that collected by each of the other four trap types. The diversity of insects we collected in one summer of trapping suggests that the Composite Insect Trap could conveniently be used as a part of biodiversity assessment or a species inventory at a given location (e.g. [23]). With the number and diversity of insect types it collects, it could provide a broad overview of the insects that live within that environment. Because the use of multiple species in several different taxa would be a more reliable indicator of ecosystem health than a single indicator, the Composite Insect Trap could be used in conservation work where the ecosystem must be assessed for overall health [26]. Similarly, insects that act as ecological indicators could help in the assessment of restoration areas [4].

In addition to evaluating the relative effectiveness of the Composite Insect Trap, we collected information from major biological retailers to compare the cost of the Composite Insect Trap to other trap types (Table 2). This simple evaluation demonstrates the relative affordability of the Composite Insect Trap. It appears that that only malaise and flight intercept traps can be ordered prefabricated, although a smaller fabric cone trap is also sold (Table 2). The “Texas” style cone trap must be built locally as it is neither commercially available, nor easily shipped. The nets for a flight intercept trap are sold without a pan, but pan traps can be made by painting any aluminum or plastic container, and their price is minimal. The pan component of flight intercept traps and stand alone pan traps are similar to the pan component of the Composite Insect Trap.

**Table 2:** Prices for the Composite Insect Trap and other commonly used trap types collected from major biological retailers.

Trap Name	Brief Description	Example Source	Estimated Cost
**Composite Insect Trap**	Flight intercept/Malaise/Cone/Pan trap composite for diversity studies	____	$77.50 US
**Malaise Trap**	Flight trap with mesh barrier and killing reagent in collection chamber above	BugDorm Store: http://bugdorm.megaview.com.tw/ or John W. Hock Company: http://www.johnwhock.com/	$184.00–$574.00 US
**Flight Intercept Trap**	Flight trap with mesh barrier and killing reagent in collection chamber below	Alana Ecology: http://www.alanaecology.com/ or Sante Traps: http://www.santetraps.com/	$30.00 US
**Pan Trap**	Dish on ground filled with killing reagent	Fleischer 2010, pers. comm	$2.00–$10.00 US
**“Texas” Cone Trap**	Large wire cone trap with pheromone bait in collection chamber above	Fleischer 2010, pers. comm	$225.00–$275.00 US
**Maryland Wire Cone Trap**	Smaller “Texas” type trap	S. Fleischer, personal communication	$180.00 US
**Scentry Heliothis Trap**	Nylon mesh cone with pheromone to attract members of the Heliothis genus	Gempler's: http://www.gemplers.com/	$80.00 US

Websites accessed April 2010.

For a study that requires multiple trap types, the supplies for the Composite Insect Trap (approximately $25.00 US) would represent a savings of up to 90%–97% (Table 2). Even after adding in the time spent constructing the insect trap, it compares favorably. Approximately 42 hours were spent constructing 6 Composite Insect Traps: an average of seven hours per trap. At a cost of $7.50 US per hour for labor, this equates to an additional $52.50 US per trap, or a price of $77.50 US per Composite Insect Trap. At this cost, the Composite Insect Trap represents a savings of up to between 70%–90%.

To purchase and employ each of the four trap types used in the design of the Composite Insect Trap would be both time consuming and expensive. Our review of some common traps available through major biological retailers suggests that the Composite Insect Trap is a much more affordable option than either the malaise trap or cone trap alone, and that its relative value is much greater when multiple trap types are considered.

If long-term monitoring is of interest, the Composite Insect Trap has the potential to simplify the sampling protocol because it combines multiple trap methods into one. In this way, it reduces the variation due to trap type and will be more easily standardized. For example, it could be incorporated into a design where the long-term monitoring of multiple pest species is an objective [13]. Eliminating the necessity for multiple traps may facilitate cross-study comparisons as well as reduce the cost and time required to implement a sampling regime.

To the best of our knowledge, the Composite Insect Trap is the only trap of its kind to combine multiple different methods to capture many different insect taxa. The Composite Insect Trap is flexible in its usage because individuals using the trap may choose to construct it to their own desired size specifications to address different ecological questions. However, its greatest utility lies in its capacity to catch a large diversity of insects. In addition, it is ideal for pilot studies, studies on a restricted budget, educational collections, and for those interested in diversity as opposed to a single taxon or guild. Because it is affordable to construct, simple to assemble, robust and easy to transport, more traps can be built within a restricted budget or time frame. The Composite Insect Trap has the potential to eliminate the need to use multiple trap types in studies of biodiversity and in the assessment of conservation and restoration areas where insects are ecological indicators.
